# Generation of a large volume of clinically relevant nanometre-sized ultra-high-molecular-weight polyethylene wear particles for cell culture studies

**DOI:** 10.1177/0954411914528308

**Published:** 2014-04

**Authors:** Aiqin Liu, Eileen Ingham, John Fisher, Joanne L Tipper

**Affiliations:** 1 Institute of Medical and Biological Engineering, School of Mechanical Engineering, Faculty of Engineering, University of Leeds, Leeds, UK; 2 Institute of Medical and Biological Engineering, School of Biomedical Sciences, Faculty of Biological Sciences, University of Leeds, Leeds, UK

**Keywords:** Ultra-high-molecular-weight polyethylene, nanoparticle, wear, simulation

## Abstract

It has recently been shown that the wear of ultra-high-molecular-weight polyethylene in hip and knee prostheses leads to the generation of nanometre-sized particles, in addition to micron-sized particles. The biological activity of nanometre-sized ultra-high-molecular-weight polyethylene wear particles has not, however, previously been studied due to difficulties in generating sufficient volumes of nanometre-sized ultra-high-molecular-weight polyethylene wear particles suitable for cell culture studies. In this study, wear simulation methods were investigated to generate a large volume of endotoxin-free clinically relevant nanometre-sized ultra-high-molecular-weight polyethylene wear particles. Both single-station and six-station multidirectional pin-on-plate wear simulators were used to generate ultra-high-molecular-weight polyethylene wear particles under sterile and non-sterile conditions. Microbial contamination and endotoxin levels in the lubricants were determined. The results indicated that microbial contamination was absent and endotoxin levels were low and within acceptable limits for the pharmaceutical industry, when a six-station pin-on-plate wear simulator was used to generate ultra-high-molecular-weight polyethylene wear particles in a non-sterile environment. Different pore-sized polycarbonate filters were investigated to isolate nanometre-sized ultra-high-molecular-weight polyethylene wear particles from the wear test lubricants. The use of the filter sequence of 10, 1, 0.1, 0.1 and 0.015 µm pore sizes allowed successful isolation of ultra-high-molecular-weight polyethylene wear particles with a size range of < 100 nm, which was suitable for cell culture studies.

## Introduction

Ultra-high-molecular-weight polyethylene (UHMWPE) wear particles have been shown to play a major role in late aseptic loosening of conventional total hip replacements (THRs).^1,2^ Recently, as a result of the high-resolution and magnification capabilities of scanning electron microscopes, nanometre-sized UHMWPE particles have been identified in both in vitro wear simulator lubricants and periprosthetic tissue samples.^3–6^ Previous studies investigating the in vitro macrophage response to UHMWPE wear particles concluded that UHMWPE particles in the size range of 0.1–1.0 µm were the most biologically active; however, particles in the nanometre-sized range were not investigated.^7–9^ It has been shown that nanometre-sized metal debris generated in metal-on-metal THR plays a role in a series of adverse tissue reactions such as tissue necrosis and pseudotumors.^10,11^ Compared to metal particles, UHMWPE is a chemically inert material and cells are not able to degrade the particles once they have been internalised, despite the low pH of the intracellular compartment. Even though the nanometre-sized UHMWPE wear particles occupy a relatively small percentage of the total wear volume, they are produced in large numbers. Due to their small size, numerous nanometre-sized UHMWPE wear particles may be disseminated throughout the body and UHMWPE wear particles have previously been found in the lymph nodes, spleen and liver.^12–14^ Therefore, it is essential to determine the relative contribution of nanometre-sized UHMWPE to the inflammatory process in osteolysis of THRs. Currently, however, there are no reports on the biological response to nanometre-sized UHMWPE wear particles, due to the difficulty in obtaining sufficiently large volumes of nanometre-sized UHMWPE wear particles for in vitro studies.

Endotoxin is a major constituent of gram-negative bacterial cell walls, which easily adheres to polyethylene particles. Endotoxin stimulates macrophages to produce nitric oxide (NO), cytokines, such as tumour necrosis factor-α (TNF-α), and interleukins (ILs), such as IL-6.^15^ It is therefore critical that any contaminating endotoxin is removed from wear particles prior to cell culture studies.

Endotoxin contamination is not only prevalent in the environment such as air, tap water and an individual’s skin, but also exists in laboratory environments, such as chemical reagents, buffers, raw materials, glassware and equipment.^16^ Endotoxin is highly heat stable, and standard autoclaving at 121 °C is not able to destroy endotoxin. It has been reported that endotoxin can be destroyed by heating to 250 °C for more than 30 min or 180 °C for more than 3 h; however, polyethylene particles that are a type of polymer material cannot withstand these conditions.^17^

Several studies have investigated different methods of removing endotoxin from UHMWPE wear particles using either chemical washing or ultracentrifugation methods.^18,19^ Although these studies provided efficient methods for removing endotoxin from UHMWPE wear particles, loss of particles after the treatment could not be avoided.

In our previous studies, it has been shown that it is possible to generate endotoxin-free, clinically relevant UHMWPE wear particles in a lubricant of RPMI 1640 supplemented with 25% (v/v) foetal calf serum using a single-station pin-on-plate simple configuration wear simulator under aseptic conditions.^20^ Therefore, generating UHMWPE wear particles in a sterile environment, which would remove any potential source of endotoxin, provided a promising way to generate endotoxin-free particles while also avoiding loss of particles. The aims of this study were to investigate different pin-on-plate simulation methods for generation of a large volume of endotoxin-free clinically relevant nanometre-sized UHMWPE wear particles suitable for cell culture studies.

## Materials and methods

### Materials

GUR 1020 UHMWPE pins (DePuy International Ltd, Leeds, UK) were sterilised using gamma irradiation in a vacuum (2.5–4 mrad). High-carbon (>0.2%, w/w) cobalt–chromium alloy (CoCr) plates (School of Mechanical Engineering, University of Leeds, Leeds, UK) had a surface roughness (Ra) of 0.07–0.08 µm.

### Methods

#### Generation of UHMWPE wear particles using a single-station pin-on-plate simulator

**Figure 1.**
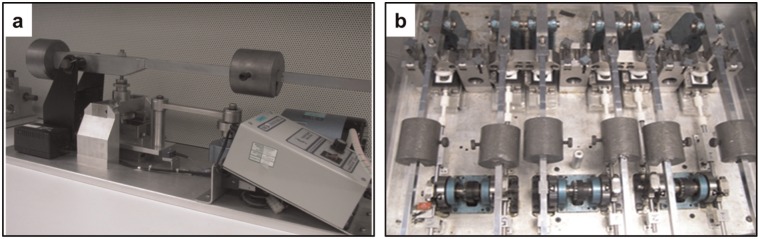
Multidirectional pin-on-plate wear simulators: (a) single-station wear rig and (b) six-station wear rig.

The multidirectional single-station pin-on-plate wear simulator was assembled using aseptic technique and run within a class II safety cabinet (Heraeus, Hanau, Germany; Figure 1(a)). Roswell Park Memorial Institute (RPMI) 1640 medium is a type of cell culture medium suitable for use with a wide variety of mammalian cells, which has been used to culture primary murine and human macrophages challenged with UHMWPE particles in previous studies.^8,20^ RPMI 1640 medium (BioWhittaker, Lonza, Verviers, Belgium) was used as a lubricant for the wear test. Metal components (stainless steel) of the wear rig and CoCr plates were sterilised by dry heat at 190 °C for 4 h, and the polymer parts (polypropylene) were sterilised by autoclaving at 121 °C for 20 min at 103 kPa pressure prior to the test. The test was run at 1 Hz, with a stroke length of 28 mm and a rotation of ±30°. A load of 160 N was applied to produce a nominal contact pressure of 3.2 MPa. The wear tests were run for a total of 14 weeks. The sterility of the RPMI 1640 medium lubricant was evaluated daily by microbiological testing. Post-test lubricants containing UHMWPE wear particles were collected and stored at −20 °C for isolation of particles.

#### Generation of UHMWPE wear particles using a six-station pin-on-plate simulator

The six-station wear testing was performed in a class II standard laboratory and covered with Perspex lid, which was a non-sterile environment (Figure 1(b)). Aseptic operation was applied where possible during assembly and operation of wear rig. The wear test was run under the same conditions as for single-station wear tests described above. Microbiological contamination of the lubricant was evaluated daily by performing microbiological tests. Post-test lubricants were stored at −20 °C until required for particle isolation.

#### Isolation and characterisation of nanometre-sized UHMWPE wear particles from wear test lubricants

Nanometre-sized UHMWPE wear particles were isolated from the wear test lubricants by filtering through descending pore-sized polycarbonate membrane filters (Whatman, Maidstone, Kent, UK). Three filter sequences were investigated, including (A) 10, 1, 0.1 and 0.015 µm filters; (B) 10, 1, 0.1, 0.05 and 0.015 µm filters and (C) 10, 1, 0.1, 0.1 and 0.015 µm filters. Prior to filtration, the wear test lubricants were subjected to a minimum of 40 min of sonication in an ultrasonic water bath (Grant Instruments (Cambridge) Ltd, Royston, Herts, UK) to limit agglomeration of wear particles. After filtration, the final 0.015 µm filters were dried under an infrared heat lamp for 4 h in a moisture-controlled room. The filters were then weighed a minimum of five times on a Mettler Toledo AT 21 digital microbalance (Leicester, UK) with a measuring accuracy of ±5 µg. The mass of the nanoscale particles was calculated.

A small section of the 0.015 µm filter was cut and mounted onto an aluminium stub and sputter coated with platinum/palladium (Agar Scientific Ltd, Stansted, UK) to a thickness of 3.0 nm, which eliminated electron charging of the particles. The particles on the 0.015 µm filters were characterised using high-resolution field emission gun-scanning electron microscopy (FEG-SEM; LEO Electron Microscopy Ltd, Cambridge, UK) images.

Particles were analysed manually using Image-Pro Plus software (Media Cybernetics, Rockville, MD, USA), and the length of the particles and their area were measured. A minimum of 100 particles were analysed for each sample using a range of magnifications from 10,000× to 200,000×. For samples with a large number of nanometre-sized particles, more than 500 particles were measured for each image. The data were exported to an Excel spreadsheet and grouped into different size ranges according to the length of the particles. The area of the image was recorded (A), and the number of particles in each size range was recorded (N). P represented the sum of the areas of the particles in each size range. Values of the number of particles per area (N/A) and the average area of particles (P/N) for each size range within each field of view were calculated. The percentage number of particles and percentage area distribution of particles in each size range were generated from these data for illustrative purposes.

Energy-dispersive X-ray analysis (EDX, INCA 350 EDX system; Oxford Instruments, Abingdon, UK) was used to determine the elemental composition of the particles of all morphologies on the 0.015 µm  filters. EDX allowed identification of contaminants; however, it is not a method that allows positive identification of UHMWPE, since the UHMWPE particles contain the elements carbon, hydrogen and oxygen, which are also present in the polycarbonate filter. Therefore, particles, which showed main element peaks including carbon and oxygen on the EDX trace, were considered to be UHMWPE.

#### Microbiological tests and endotoxin tests

Microbiological tests were performed by plating lubricant samples on heated blood agar (HBA), nutrient agar (NA) and Sabouraud dextrose agar (SAB). The HBA and NA plates were incubated at 37 °C, and the SAB plates were incubated at 30 °C for 24 h. A volume of 1 mL of lubricant sample was transferred into 10 mL of nutrient broth and incubated with shaking at 37 °C for a minimum of 2 weeks. All microbiological culture media were supplied by the Media Lab, Institute of Molecular and Cell Biology, University of Leeds, Leeds, UK.

The pyrochrome Limulus amebocyte lysate (LAL) kinetic-QCL endotoxin assay (Associates of Cape Cod, Incorporated, East Falmouth, MA, USA) was used to determine the presence of endotoxin in the wear test lubricants and fractionated nanometre-sized particle stocks. The assay was performed according to the manufacturers’ instructions.

## Results

### Characterisation of UHMWPE wear particles generated from single-station wear simulation

**Figure 2.**
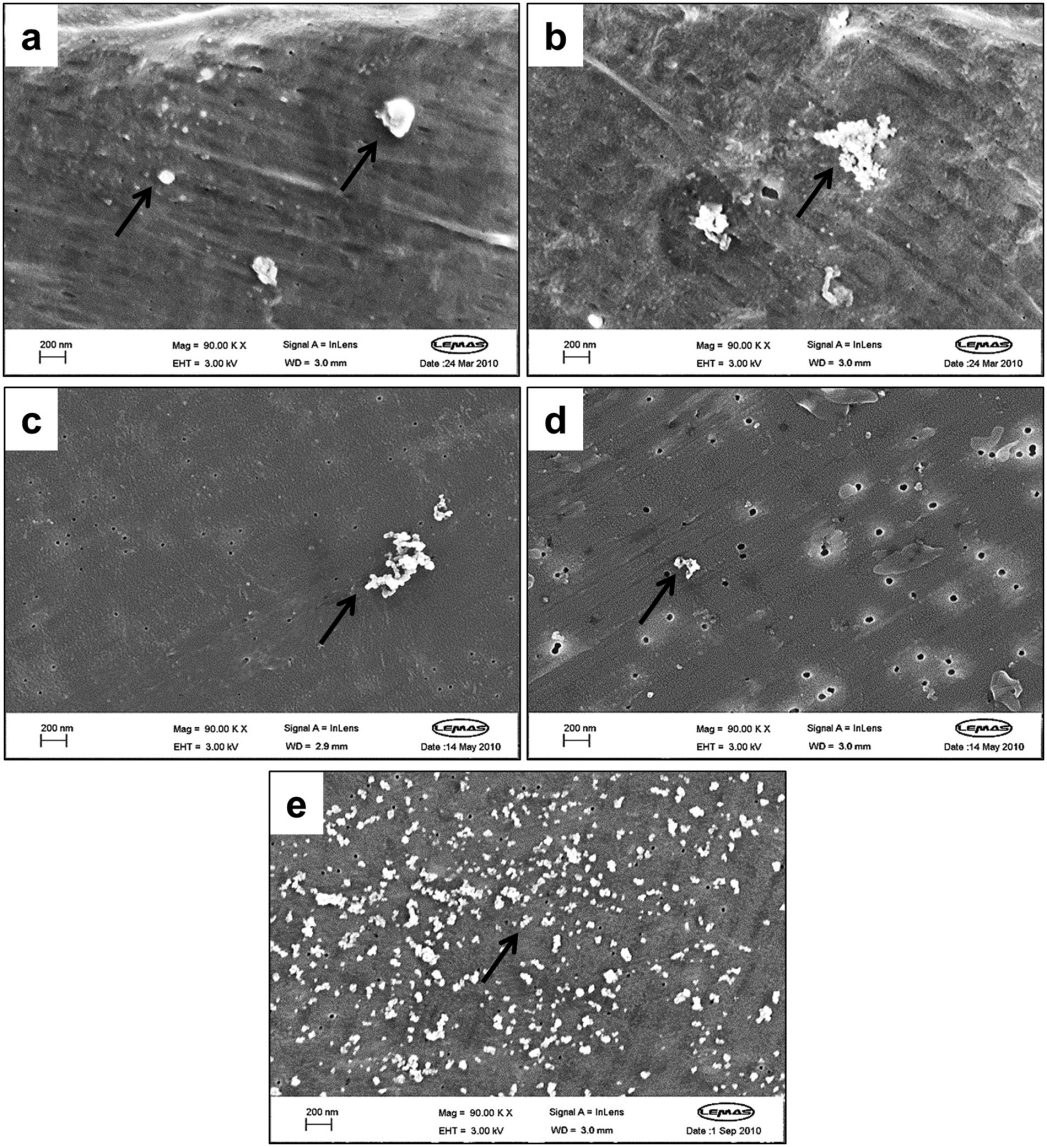
FEG-SEM images of UHMWPE wear debris (indicated by arrows) isolated from lubricants from single-station wear simulations: (a) micrometre-sized wear particles on the 0.015 µm filter isolated from filter sequence A (10, 1, 0.1 and 0.015 µm filters), analysed at 90,000×; (b) agglomerated nanometre-sized granular wear particles on the 0.015 µm filter from filter sequence A (10, 1, 0.1 and 0.015 µm filters), analysed at 90,000×; (c) agglomerated nanometre-sized granules on the 0.015 m filter from filter sequence B (10, 1, 0.1, 0.05 and 0.015 µm filters), analysed at 90,000×; (d) agglomerated granular particles observed on the 0.05 µm filter from filter sequence B (10, 1, 0.1, 0.05 and 0.015 µm filters), analysed at 90,000× and (e) agglomerated nanometre-sized particles on the 0.015 µm filter from filter sequence C (10, 1, 0.1, 0.1 and 0.015 µm filters), analysed at 90,000×.

The lubricants obtained from the first 3 weeks of wear testing were filtered through a filter sequence A (10, 1, 0.1 and 0.015 µm filters), and particles were collected on the 0.015 µm filter. The morphologies of particles isolated from filter sequence A are shown in Figure 2(a) and (b). Irregular shaped and granule type particles, with a size greater than 100 nm, were commonly observed on the 0.015 µm filter (Figure 2(a)). Agglomerated nanometre-sized particles with a granular shape were observed in abundance (Figure 2(b)). A 0.05 µm filter was added before the 0.015 µm filter during the filtration in order to reduce the percentage area of particles with a size of greater than 100 nm. Therefore, the lubricants obtained from the following 3 weeks of wear testing were filtered through the filter sequence B (10, 1, 0.1, 0.05 and 0.015 µm filters). After the addition of the 0.05 µm filter into the filter sequence B, larger particles (>100 nm) were not observed on the 0.015 µm filter (Figure 2(c)), and the majority of the particles observed were within the nanometre size. However, agglomerated nanometre-sized particles were also commonly observed on the 0.05 µm filter (Figure 2(d)). The lubricants obtained from the following 8 weeks of wear testing were filtered through filter sequence C (10, 1, 0.1, 0.1 and 0.015 µm filters), and a large number of nanometre-sized particles were recovered on the 0.015 µm filter (Figure 2(e)). The particles were in the size range of less than 100 nm, with the majority of the particles being less than 50 nm. The total weight loss for the 8 weeks was 256.7 mg; however, a mass of only 47 µg of nanometre-sized UHMWPE wear particles was obtained from lubricants filtered through filter sequence C.

**Figure 3.**
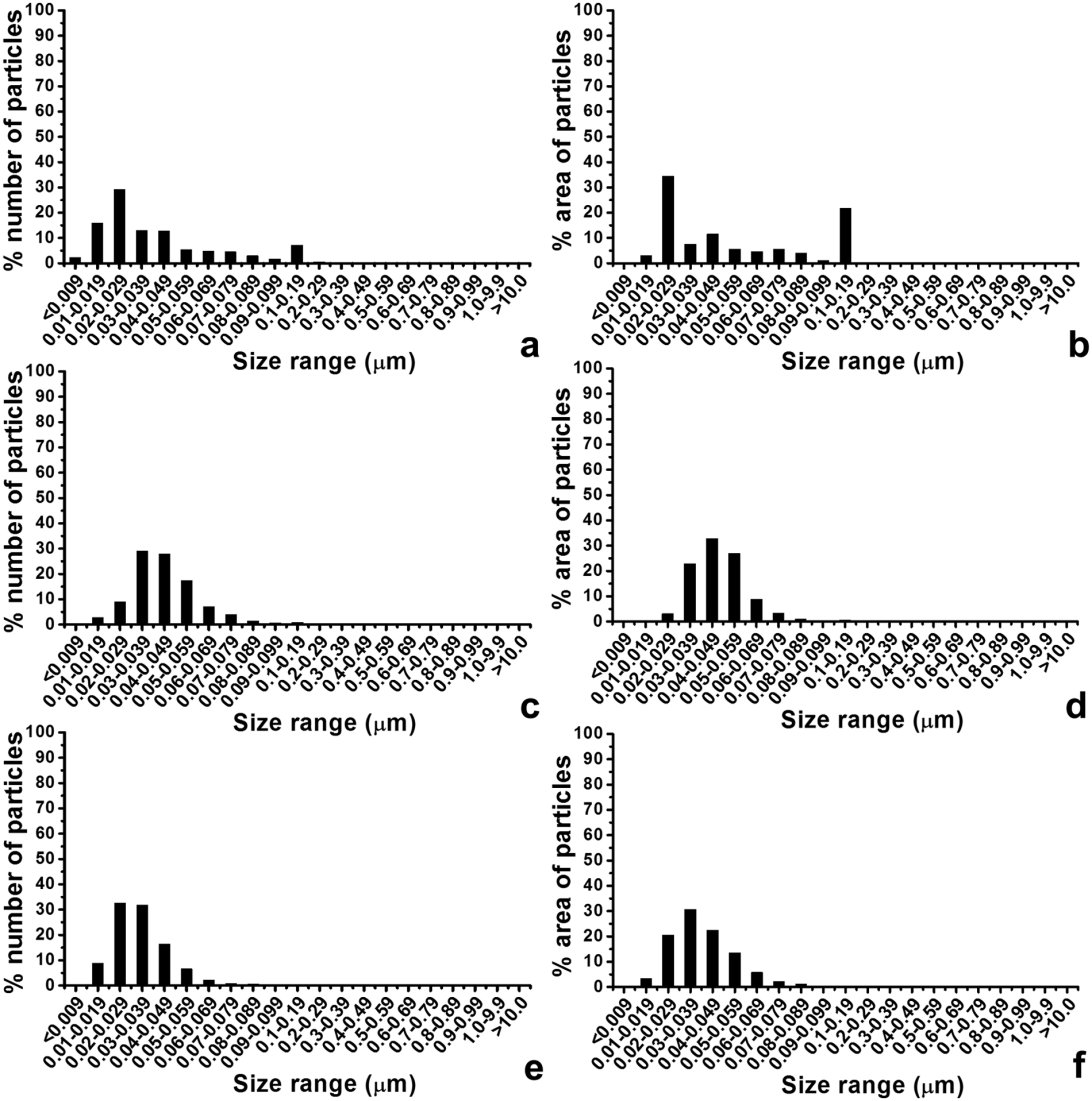
Percentage number and the percentage area distributions as a function of size of nanometre-sized wear particles from three filter sequences: (a, b) filter sequence A, (c, d) filter sequence B and (e, f) filter sequence C.

For particles isolated from filter sequence A, although 90.19% of the particles were less than 100 nm in size, particles with a size range of greater than 100 nm accounted for a high percentage area of particles (22.07%; Figure 3(a) and (b)). For particles from filter sequence B, the percentage number and the percentage area of particles in the size range above 100 nm were decreased to 0.84% and 0.54%, respectively (Figure 3(c) and (d)). For particles from filter sequence C, only 0.074% of the total number of the wear particles was greater than 100 nm in size (Figure 3(e)). The area percentage of the particles with a size greater than 100 nm was reduced to 0.048% (Figure 3(f)).

### Characterisation of UHMWPE wear particles generated from six-station wear simulation

**Figure 4.**
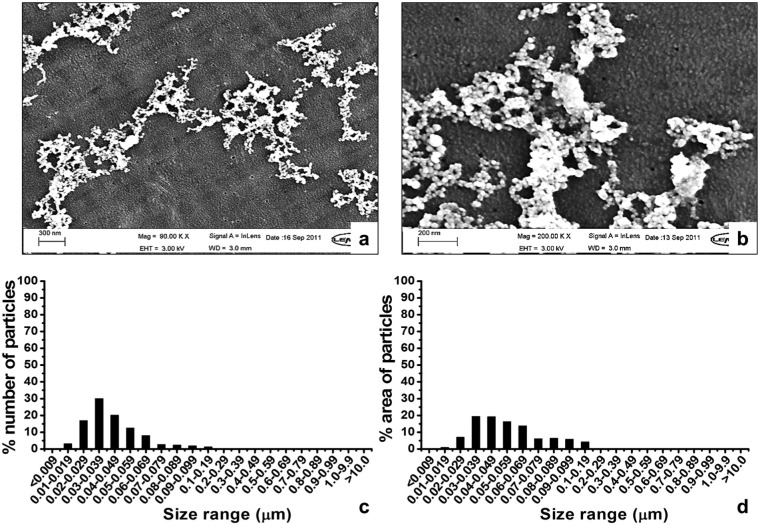
FEG-SEM images and size distributions of UHMWPE wear debris isolated from lubricants from six-station wear simulation by filtering through a filter sequence of 10, 1, 0.1, 0.1 and 0.015 µm filters: (a) nanometre-sized wear debris analysed at 90,000×, (b) nanometre-sized wear debris analysed at 200,000×, (c) the percentage number and (d) the percentage area distributions of wear debris.

The lubricants from six-station wear simulation were filtered through the filter sequence C (10, 1, 0.1, 0.1 and 0.015 µm pore-sized filters). The morphologies of particles obtained on the final 0.015 µm filters are shown in Figure 4. Agglomerated granular nanometre-sized particles were abundant on the 0.015 µm filter (Figure 4(a)). Particles with a size range greater than 100 nm were occasionally seen on the filter. Particles with a size range less than 50 nm were observed clearly at a high magnification of 200,000× (Figure 4(b)). Of the wear particles isolated on the 0.015 µm filter, 99.2% were in the nanometre-sized range of less than 100 nm (Figure 4(c)). The mode of the percentage number of particles was 30–39 nm. Particles in the less than 100 nm size range accounted for the majority of the area on the 0.015 µm filter, while the percentage area represented by particles with the size of above 100 nm was 4.5% (Figure 4(d)).

### Bacterial and endotoxin levels in simulator lubricants and isolated particles

All of the wear tests performed in the single-station and six-station pin-on-plate simulators were free from microbiological contamination. The six-station lubricants and isolated nanometre-sized particle stocks showed very low endotoxin levels, which were below the accepted value of less than 5 endotoxin units (EU) according to the specification of endotoxin tolerance limit for non-pyrogenic products for the pharmaceutical industry.^21^

### EDX analysis of polyethylene particles on 0.015 µm filters

**Figure 5.**
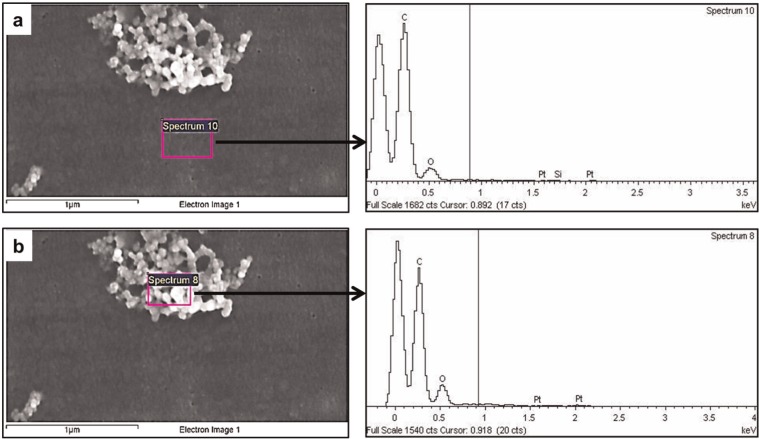
SEM images and EDX traces of (a) 0.015 µm polycarbonate filter and (b) nanometre-sized particles. The square on the image indicates the area where the EDX spectrum was detected.

The main peaks for the polycarbonate filter were carbon and oxygen. Trace elements of platinum and silicon were also present at very low levels, as shown in Figure 5(a). The trace of platinum on the spectrum indicated the presence of the coating. The dominant peaks on the spectrum detected from an area of typical agglomerated particles belonged to the elements of carbon and oxygen (Figure 5(b)), which were very similar to those of the polycarbonate filter (Figure 5(a)). No contaminant traces were identified within the particles detected (Figure 5(b)). Therefore, the particles were considered to be UHMWPE, which indicated that the large number of nanometre-sized particles on the 0.015 µm filter were UHMWPE wear particles.

## Discussion

The prefix ‘nano’ is used in science to mean 1 × 10^−9^. Particles that are less than 100 nm in size have been defined as being in the nanometre-sized range in previous studies.^3–6^ Therefore, the aim of this study was to generate only nanometre-sized UHMWPE wear particles, that is, those smaller than 100 nm, in isolation.

The size and volume concentration of particles have been shown to be the most important determinants in cell responses to UHMWPE particles,^22^ while other factors, such as molecular weight, composition, surface texture and shape, have also been shown to stimulate different cellular responses.^20,23,24^ Previous studies^18,25,26^ have used commercially available UHMWPE particles, which were convenient and cost-effective; however, the particles were almost spherical in shape and had quite different morphologies from particles isolated from retrieved tissues of patients with failed total hip prostheses, which were more varied in shape and had irregular surface textures. Therefore, it is important to generate particles by wear simulation to ensure that they are representative of clinically generated particles.

Generating large volumes, sterile, clinically relevant particles were three key problems in this study. The single-station pin-on-plate simulator was used initially to generate clinically relevant nanometre-sized UHMWPE wear particles under aseptic conditions. Microbiological tests showed that none of the wear tests experienced bacterial contamination. In order to obtain particles with a size of less than 100 nm, three different filtration sequences were investigated. Filter sequence C, which comprised 10, 1, 0.1, 0.1 and 0.015 µm filters, generated the lowest percentage area of particles greater than 100 nm (0.048%) and produced particles only within the nanometre-sized range (<100 nm). This filter sequence represented the optimised size distribution for isolating nanometre-sized UHMWPE wear debris. Furthermore, the agglomerated granular morphologies of the nanometre-sized UHMWPE particles isolated were extremely similar to those from retrieved tissues,^5,6^ which indicated that the nanometre-sized UHMWPE wear particles generated in this study were clinically relevant particles. The use of the single-station wear simulators solved two of the problems in generating clinically relevant sterile particles; however, a mass of only 47 µg of nanometre-sized particles, which was equivalent to 0.0183% (w/w) of the total wear loss, was obtained after 8 weeks of wear testing, indicating that it was not practical to generate a large volume of UHMWPE wear particles using the single-station wear simulator.

Therefore, the feasibility of using a six-station multidirectional pin-on-plate wear simulator was investigated, which was expected to produce six times the amount of wear compared to the single-station wear simulator. As the six-station wear simulator could not be housed in a sterile environment, it was a challenge to generate endotoxin-free wear particles in a non-sterile environment. However, as a result of careful set-up and operation and the omission of serum from the RPMI 1640 medium lubricant, there was no microbiological contamination of the lubricants, and the isolated nanometre-sized UHMWPE wear particles showed acceptable endotoxin levels, which indicated that it was feasible to generate endotoxin-free wear particles in a non-sterile environment.

Tipper et al.^4^ reported, for the first time, on the isolation of nanometre-sized UHMWPE particles down to 10 nm from the lubricants retrieved from hip and knee simulators. Richards et al.^5^ identified nanometre-sized UHMWPE particles from tissue samples taken from failed Charnley THRs. The smallest particle identified in vivo was 30 nm. Recently, Lapcikova et al.^6^ isolated nanometre-sized UHMWPE wear particles with a size of less than 50 nm from periprosthetic (granuloma) tissues obtained during revision of total joint replacements. In this study, nanometre-sized wear particles with a size range below 10 nm were identified in the lubricants from six-station pin-on-plate wear simulation (Figure 4(b)). The mode size of the particles isolated was 30–40 nm, which was consistent with the size range of nanometre-sized particles identified in vivo.^6^

The total weight loss for 8 weeks of six-station testing was 1388.12 mg, while those for 8 weeks of single-station testing was 256.7 mg, which indicated that the production of wear particles was increased using six-station wear test. Most importantly of all, a stock of 1225 µg nanometre-sized particles was obtained after 8 weeks of wear testing (equivalent to 0.0882% (w/w) of the total wear loss), which indicated that the use of the six-station multidirectional pin-on-plate wear simulator achieved the aim of obtaining a large volume of sterile clinically relevant UHMWPE wear particles, which would be suitable for cell culture studies to investigate the biological response to nanometre-sized UHMWPE wear particles.

## Conclusion

A multidirectional pin-on-plate six-station wear simulator provided an efficient way to generate a large volume of endotoxin-free, clinically relevant nanometre-sized UHMWPE wear particles in vitro in order to investigate the contribution of nanoscale wear debris to osteolysis inflammation and adverse biological responses.
